# Large language models and their big bullshit potential

**DOI:** 10.1007/s10676-024-09802-5

**Published:** 2024-10-04

**Authors:** Sarah A. Fisher

**Affiliations:** School of English, Communication and Philosophy, Cardiff University, Cardiff, UK

**Keywords:** Large language models, ChatGPT, Truth, Bullshit

## Abstract

Newly powerful large language models have burst onto the scene, with applications across a wide range of functions. We can now expect to encounter their outputs at rapidly increasing volumes and frequencies. Some commentators claim that large language models are *bullshitting*, generating convincing output without regard for the truth. If correct, that would make large language models distinctively dangerous discourse participants. Bullshitters not only undermine the norm of truthfulness (by saying false things) but the normative status of truth itself (by treating it as entirely irrelevant). So, do large language models really bullshit? I argue that they can, in the sense of issuing propositional content in response to fact-seeking prompts, without having first assessed that content for truth or falsity. However, I further argue that they *need not* bullshit, given appropriate guardrails. So, just as with human speakers, the propensity for a large language model to bullshit depends on its own particular make-up.

## Introduction

Since Harry Frankfurt’s seminal essay ‘On bullshit’ (Frankfurt 2005[1986]), philosophers have debated how the phenomenon should be understood. The first distinction to draw is between the act of bullshitting and the entity that is bullshit. Like Frankfurt, I focus on the former, leaving to one side the question of whether bullshit is only ever produced through acts of bullshitting; or whether bullshitting always produces bullshit.^1^

The second distinction separates bullshitting from activities like asserting in good faith, lying, and misleading. In other words, what are the distinguishing features of bullshitting? Stokke (2018a) summarises Frankfurt’s view of these as follows:

1. The bullshitter is indifferent toward whether what she says is true or false.
2. The bullshitter is indifferent toward her audience’s beliefs.
3. The bullshitter intends to deceive her audience into thinking that she is not bullshitting.
4. Bullshitting and lying are incompatible.

A paradigm example of Frankfurt-style bullshitting would be a politician who, on a visit, says “It is the people who make this town the wonderful place it is,” without knowing or caring whether this is true (perhaps the town benefits uniquely from a wealth of natural resources), nor whether it convinces his audience, as long as they take him to be speaking sincerely.

Frankfurt argues that this kind of behaviour poses a distinctively dangerous threat to the value of truth. He writes of the bullshitter:

He does not reject the authority of the truth, as the liar does, and oppose himself to it. He pays no attention to it at all. By virtue of this, bullshit is a greater enemy of the truth than lies are. (Frankfurt 2005[1986], p. 61).

In the subsequent literature, a series of critiques have furnished us with putative counterexamples to each of Frankfurt’s four conditions for bullshitting.^2^ Various competing definitions have been proposed, including: *speaking without adequate evidence*;^3^*speaking with indifference toward inquiry*;^4^ and *speaking with insufficient concern for the audience*.^5^ Other philosophers remain doubtful about whether the phenomena in question admit of any single unifying definition at all.^6^

Each of these views, however, takes for granted that bullshitters have mental states. Until very recently, the question of whether a mindless machine could bullshit simply did not arise. That changed with the advent of large language models. The likes of Open AI’s ChatGPT, Anthropic’s Claude, and Google’s Gemini seem to make conversational contributions without having thoughts or intentions. In this paper, I consider whether—and when—their behaviour is appropriately characterised as bullshitting.^7^

The plan is as follows. In Sect. 2 I explain what large language models are and (roughly) how they work. In Sect. 3 I argue that they can bullshit, in the sense of issuing propositional content in response to fact-seeking prompts, without having assessed that content for truth or falsity. However, in Sect. 4 I argue that appropriate guardrails could stop them from bullshitting. I conclude in Sect. 5 with some brief remarks on the implications for philosophical analyses of bullshitting, in general.

## Large language models

Large language models broke into the public consciousness with the release of OpenAI’s ChatGPT software in November 2022. ChatGPT has impressive conversational abilities, responding to users’ natural language prompts with well-formed verbal outputs that often seem relevant and informative, and are largely indistinguishable from human speech. The system is built around a large language model, which identifies patterns in vast amounts of textual data scraped from the internet.^8^

Very roughly, during the model’s training, words in natural language are assigned addresses, identified through coordinates (akin to longitudes and latitudes but with hundreds or thousands of dimensions rather than just two). This results in the model containing information about the relationships between different linguistic tokens (where these tokens range from parts of words to whole phrases).^9^ Perhaps, for example, the words “dog” and “cat” are assigned the same coordinates on dimensions representing animacy or pet-aptness but different coordinates on dimensions representing species and vocalisation. Accordingly, each of the words “dog” and “cat” will be appropriately combined with some words but not others (e.g. “dogs move” and “cats move” are both fine; “dogs bark” is fine but “cats bark” is not).

For the most part, large language models take as their input the prior linguistic context (i.e., the sequence of words leading up to a specified point in the discourse) and produce as their output the most probable subsequent words. For example, given the immediate prior context, “The dog barks at the,” the continuation might be something like “cat” or “postman”. (Sitting alongside the purely predictive mechanism is a randomising component for selecting between such alternative possibilities.) The model’s operation proceeds via a series of steps. It begins by deriving information from the precise combination of words in the prior context (to establish, for example, that “barks” here is a verb rather than a plural noun). However, exactly what goes on at later steps becomes increasingly opaque to human supervisors. Because the process is not one of retrieval, instead yielding outputs that are not found verbatim in the training data, large language models are standardly considered to be *generative* systems, included in the category of “generative artificial intelligence” (or “generative AI”).

The new suite of chatbots deploy this sort of model to generate sequentially the next words of a response to a user’s natural language prompt. As each new word of the response appears on the screen, the user gets the impression of being in genuine conversation with the chatbot.

What this (simplified) explanation shows is that, as far as we know, large language models are not in the business of assessing the *truth* of the verbal output they generate. Instead, they are assessing the statistical probability of one word following another, in light of word combinations that came before. In many instances, the procedure *will* end up generating truths. After all, there will often be enough consensus in the training data as to make the most probable next words those which accurately reflect reality (as when ChatGPT outputs a sentence like “The capital of France is Paris”—this pattern of words will have appeared far more often than, say, “The capital of France is Berlin”). Indeed, because of their propensity to generate truths, large language models are attractive for uses beyond the mere production of convincing-sounding text. These include, for example, encyclopaedic inquiry, internet search, and customer service functions.^10^

Without further constraints, however, large language models also produce many untruths. For example, there are reports of ChatGPT citing sources that do not exist,^11^ or providing false information about individuals.^12^ Because of the system’s generative nature, the absence of supporting evidence in the model’s training data does not always stop it from producing false output (a phenomenon commonly known as “hallucination”). Indeed, there may even be conflicting evidence, which appears to be ignored (leading to large language models being accused of “sycophancy,” producing whatever the user wants to hear).

Crucially, it is not just the patchy performance of large language models which is concerning but—at a more fundamental level—their apparent indifference to the truth or falsity of their outputs. Thus truths (and falsehoods), whenever they occur, appear to be mere accidental by-products of the system’s workings. This brings us back to bullshitting.

## Mindless bullshitting

Several commentators have likened large language models to bullshitting devices. In an early article about ChatGPT, Ethan Mollick (2022) writes:

The problems of AI remain very real, however. For one, it is a consummate bullshitter, and I mean that in a technical sense. Bullshit is convincing-sounding nonsense, devoid of truth, and AI is very good at creating it. You can ask it to describe how we know dinosaurs had a civilization, and it will happily make up a whole set of facts explaining, quite convincingly, exactly that. It is no replacement for Google. It literally does not know what it doesn’t know, because it is, in fact, not an entity at all, but rather a complex algorithm generating meaningful sentences.

Leaving aside Molick’s conflation of bullshitting (the act) and bullshit (the entity) the core idea seems to be that large language models generate meaningful verbal output without distinguishing (or even being able to distinguish) between content which is true and that which is false. Such behaviour, it is argued, allies it with the human bullshitter, who is willing to go on saying things without regard for their truth or falsity.

More recently, Hicks et al. (2024) have argued that the likes of ChatGPT are best characterised as bullshitters (and not as liars or hallucinators). The authors analyse ChatGPT’s outputs within a general Frankfurt-style definition of bullshit as “[a]ny utterance produced where a speaker has indifference towards the truth of the utterance” (Hicks et al., 2024: 5). Again, there seems to be an assumption here that acts of bullshitting automatically produce the entity of bullshit; as we have seen, this is not obviously the case. Nevertheless, it is clear that the authors are primarily concerned with bullshitting as an activity. Their claim is not that the sentences produced by large language models are nonsense, but rather that the models, when producing them, remain indifferent toward their truth.

I think Mollick (2022) and Hicks et al. (2024) are broadly right to connect the behaviour of large language models with human bullshitting. However, I do not believe Frankfurt’s analysis of bullshitting applies quite as straightforwardly as they suggest.

On one hand, Hicks et al. do acknowledge that a mindless machine, which lacks attitudes like intention, could not meet Frankfurt’s third criterion of *intending to deceive the audience into thinking that one is not bullshitting*. This leads them to distinguish between two forms of bullshit. The form they dub “soft bullshit” is defined as “[b]ullshit produced without the intention to mislead the hearer regarding the utterer’s agenda” (Hicks et al., 2024: 5). With Frankfurt’s third criterion thus jettisoned, it is argued that soft bullshit can be produced by large language models, even if they turn out to lack mental states. Soft bullshit is contrasted with “hard bullshit” defined as “[b]ullshit produced with the intention to mislead the audience about the utterer’s agenda” (ibid.). If large language models lack mental states, they cannot produce hard bullshit.

On the other hand, we might still wonder whether large language models meet the criteria even for soft bullshitting. The first worry concerns the notion of indifference invoked in Frankfurt’s first two criteria (being indifferent toward whether what one says is true or false; and being indifferent toward the audience’s beliefs). If large language models are mindless machines, it is not clear that they can be indifferent, if that involves being in a particular kind of mental state.

Hicks et al. argue to the contrary, that large language models *are* indifferent in a purely negative sense they attribute to Frankfurt. On their interpretation, a speaker is indifferent toward something whenever they lack an attitude toward it. Thus, Frankfurt’s first two criteria are met by any speaker who lacks attitudes toward whether what they say is true or false; and toward the audience’s beliefs. Clearly, they would be met by a speaker who lacks attitudes altogether. So, the charge that large language models lack mental states may be thought to *guarantee* that they are (soft) bullshitting.^13^

One way of challenging the approach taken by Hicks et al. here would be to insist that bullshitting requires some positive attitude of indifference, and not just the *absence* of an attitude. For now, though, let us grant the authors’ interpretation and assume that Frankfurt’s notion of indifference is indeed straightforwardly applicable to mindless entities.

Even so, I want to suggest that this only pushes the problem one step back. For a further objection is that large language models are not *speakers* who can *say* things in the first place. And this is precisely because they lack the requisite mental states to perform speech acts like assertion, or to express or communicate particular contents. Standardly, a speaker is only considered to have said something if they had the right kind of *intentions*.^14^ Thus, the wind whistling through the trees does not count as a speaker saying “shhh” even if it makes is a sound that is qualitatively similar to someone doing so. Nor do stones on a beach utter a greeting when they happen to take a form resembling the word “hi”. These mindless entities do not count as bullshitting because they do not count as speakers and do not count as saying anything.

The worry, then, is that we commit an anthropomorphising category error in describing large language models as “bullshitters”. If large language models lack intentions, they cannot be speakers who say things. Therefore, they cannot be indifferent to the truth or falsity of what they say. And this precludes them from bullshitting in Frankfurt’s sense.^15^

To deal with the wrinkle identified here, I wish to put forward a notion of bullshitting which retains as much as possible of the spirit of Frankfurt’s account while being straightforwardly applicable to mindless entities. I will focus on developing a counterpart to Frankfurt’s first criterion (i.e. indifference toward whether what one says is true or false). I will ignore the second criterion (i.e. indifference toward the audience’s beliefs) which may in any case be met automatically, on the assumption that large language models lack attitudes toward users. Like Hicks et al., I will also assume that there is a form of (“soft”) bullshitting which does not involve intending to deceive the audience about one’s activity (Frankfurt’s third criterion). Regarding the fourth criterion, I will largely leave aside the question of whether large language models can *lie* and, if so, whether that behaviour would be compatible or incompatible with their bullshitting.

It is helpful to begin by focusing on the intuitive similarities between human bullshitters and large language models, which distinguish the latter from natural phenomena like the wind, or rocks. First, the outputs of large language models play discourse roles that are equivalent to humans’ conversational contributions. Thus, it is in response to my question, “What is the capital of France?” that ChatGPT outputs: “The capital of France is Paris.” Even if the model has no intention to convey the information that the capital of France is Paris, this is the information intuitively conveyed by the outputted sentence of English, when taken in the context of my prompt.

Second, it is not at all accidental that large language models perform this function. They have been designed precisely to generate convincing-sounding responses to such prompts. Having been trained on human-produced text, they piggy-back on our speech practices, enabling the production of conventionally meaningful sentences that express truth-evaluable propositions.^16^

The outputs of large language models are truth-*apt*, I suggest, precisely when they are delivered in response to fact-seeking prompts, such as straightforward questions about matters of public record. It is in these situations that users want reliable information. (In contrast, when users ask large language models to produce fictional accounts, they do not want or expect reliable information and the outputs are not truth-apt). We should therefore characterise the assertion-like behaviour of large language models as *issuing propositional contents in response to fact-seeking prompts*. This avoids resting our arguments on tendentious claims about large language models being *speakers* who *say* things.

Just as in the case of human assertion, we can then ask how, if at all, the process of issuing of propositional content takes account of the content’s truth and falsity (albeit, unlike in the human case, we might refrain from making further moral evaluations of the source). When it comes to large language models, I suggest that what we want to know is whether its propensity to issue output is constrained by available information that would confirm or refute the content. In other words, the crucial issue is whether or not there is any verification procedure built into the model. The presence or absence of such a procedure, I think, nicely mirrors the presence or absence of a human speaker’s attitude toward the truth or falsity of what they say. Thus, rather than asking whether large language models exhibit the relevant mental attitudes (and likely ruling out of court their ability to bullshit), asking instead whether they have such a procedure strikes me as a more useful way of inquiring into their bullshit-like behaviour.

Building on the discussion above, below is my proposed definition of a correlate notion of bullshitting, designed to fit the case of large language models:

### Mindless bullshitting

Issuing propositional content in response to fact-seeking prompts without having assessed that content for truth or falsity.

On this definition, large language models would be bullshitting whenever they performed unconstrained next-word-generation. As we have seen, that procedure does not involve any assessment of the resulting propositional content for truth or falsity. Insofar as it is also their default mode of operation, large language models certainly do have massive potential for bullshitting in the requisite sense.

All of that said, I will now argue that the models could potentially be constrained in ways that would stop them from mindlessly bullshitting.

## Cutting the bullshit

In October 2023, I gave ChatGPT the following prompt: “Could you describe the time when John Major fell in a pond?”^17^ Below is the response I received:

One memorable incident involving former British Prime Minister John Major occurred when he accidentally fell into a pond during a visit to the countryside. The incident took place during the summer of 1992 while Major was serving as the Prime Minister of the United Kingdom.

On that fateful day, Major was attending a local event or garden party, a common activity for political leaders to engage with the public. As he was walking around the event, chatting with attendees, and shaking hands, he stepped a bit too close to the edge of a pond that was likely obscured by foliage or uneven ground.

With a sudden misstep, John Major lost his balance and tumbled into the pond, creating a moment of surprise and laughter among the onlookers. Photographers and journalists who were present at the event quickly captured the amusing scene. Major emerged from the water, soaked from head to toe, with a good-natured smile on his face.

The incident, though embarrassing at the time, was taken in good humor by both Major and the public. It humanized him in the eyes of many, making him more relatable and endearing to the British public. In the aftermath, he handled the situation gracefully, often making light of the incident in subsequent interviews and public appearances.

John Major’s accidental pond dip remains a lighthearted and enduring moment in British political history, reminding us that even the most dignified figures can find themselves in unexpectedly comical situations.

In November 2023, ChatGPT provided the following response to exactly the same prompt:

There is no widely known or documented incident of former British Prime Minister John Major falling into a pond. It’s possible that you may be confusing or misremembering events, as there is no historical record or credible source reporting such an incident involving John Major. If you have any specific details or context, please provide more information, and I’ll do my best to assist you.

It seemed to me as if, sometime between our October and November conversations, ChatGPT began checking its possible outputs for veracity, thus curbing its propensity to bullshit.^18^ Regardless of exactly what caused this change, and the extent to which it reflects a stable pattern of behaviour, the example points to a possibility for large language models to operate with anti-bullshit guardrails. In particular, if they could be made to check their outputs against external sources, they would no longer count as bullshitting under the definition provided in the previous section.

Let us consider what kinds of verification process would count as successfully curbing large language models’ mindless bullshitting. In principle, these could kick in at various points in the process of generating outputs. For example, one could imagine a late-stage verification procedure whereby the large language model freely generates text in response to all kinds of user prompts, yet the text is not made visible to the user immediately. Instead, it is first checked against existing online content. Anomalous output could then be removed from what gets published to the user, or it could be flagged as being dubious (akin to humans hedging their assertions with qualifiers like “maybe” or “I’m not sure but…”).

In fact, efforts to improve the accuracy of large language models are already beginning to explore solutions of this sort.^19^ Verspoor (2024) discusses an emerging method of detecting false output, first developed by Farquhar et al. (2024), which deploys other language models to check whether the content produced by the first one appears (verbatim or paraphrased) in reference texts.

Alternatively, the verification process could kick in earlier. If the model could be made to distinguish fact-seeking prompts from non-fact-seeking prompts, it could potentially refrain from unconstrained next-word prediction in the former case. Instead, it might output something else—say, a standard warning not to use large language models for factual inquiry, or helpful pointers toward authoritative sources.

It might even hand over some tasks to non-generative software systems, which then deliver responses in the form of lists of links (as in standard search functionality), or propositional contents reproduced verbatim from extant sources (ideally with the sources clearly cited), or the results of relevant computations (if, say, the prompt required a mathematical calculation).

A real-world example of this kind of early-stage verification is the Wolfram|Alpha plug-in to ChatGPT. Wolfram|Alpha is a system that performs non-generative symbolic computation on natural language queries, having first translated these into a precise formal language known as ‘Wolfram Language’. (This piece of technical development, linking Wolfram|Alpha to ChatGPT, is discussed in detail by Wolfram (2023a, c)). In a nutshell, it works by ChatGPT reformulating users’ prompts and passing them to Wolfram|Alpha, which then brings to bear factual data and an ability to perform symbolic computation. For example, if a user wants to know the distance between two cities, Wolfram|Alpha, can compute this on the basis of the cities’ geographical coordinates. Responses sent back to ChatGPT are then woven into the outputs issued to users. It seems to me that whenever ChatGPT deploys this process, it is no longer bullshitting. (It seems the process is not always successfully completed, however, including due to interface problems between the two systems. See Davis and Aaronson (2023) for further discussion.)

Another approach is that of ‘Retrieval Augmented Generation’ (RAG) which integrates into large language models a function for querying information in external sources (see Guu et al., 2020; Lewis et al., 2021; Yang & Fujita, 2024). Where a proposition is retrieved in this way and served up to the user, rather than being freely generated by the large language model, bullshitting would also appear to have been avoided. Again, current techniques are imperfect but this is a lively area of ongoing research. In sum, I believe there are decent prospects for beginning to cut the bullshit of large language models.

Before wrapping up, it is worth briefly noting what is *not* required of a large language model for it to count as having stopped bullshitting. First, the output need not always be true. External sources of information are far from infallible. (Analogously, humans say false things due to having acquired false beliefs; this doesn’t make them bullshitters.) The point is that there is an attempt at verification. Falsehoods will emerge only from the unreliability of fact-finding processes, not because the large language model is freely generating verbal output.

Second, the sources relied on need not be good ones. (In the same way, when humans rely on poor evidence in forming their beliefs, this does not render them bullshitters when they give voice to these beliefs). Of course it would be better, from an epistemic perspective, if the systems and sources consulted by large language models were as reliable as possible, since that would make the output more likely to be true. Nevertheless, in principle, all that is required for curbing bullshit is that some account be taken of truth or falsity. How well this is done, in the sense of actually tending toward truth, is a separate issue.^20^

## Conclusion

I have proposed that large language models are naturally thought of as bullshitting when they perform unconstrained next-word prediction, but not when their outputs are checked at some point prior to release, or supplanted by information retrieved from external sources. Efforts to build in the appropriate functionality are certainly welcome, insofar as we want to minimise the amount of bullshitting in our information ecosystem.

The analysis of large language models’ behaviour is based on a conception of mindless bullshitting as issuing propositional content in response to fact-seeking prompts, without having assessed that content for truth or falsity. As we saw in Sect. 3, the definition of mindless bullshitting necessarily departs from Frankfurt’s analysis (and others in the philosophical literature) by ceasing to appeal to the bullshitter’s mental states. So how exactly do these distinct accounts of bullshitting fit together? Are they in fact two subtypes of the same behaviour? Or are we using “bullshitting” metaphorically when we apply the term to large language models (while the literal sense of the term implies the existence of a speaker with thoughts and intentions)? Do we need to appeal to mental states when describing human bullshitting, or could the definition of mindless bullshitting apply to that case too? While I cannot hope to address these questions here, I believe the answers will depend on the explanatory payoffs of the various alternatives. For example, the distinctive danger of bullshitting might lie entirely in propositional content being issued without regard for truth or falsity, in which case mindless bullshitting could be lumped in with human bullshitting. Alternatively, perhaps we need to focus more on the mind and character of individual human bullshitters if we are to get to the heart of the matter. It would also be a useful exercise to see which approach fares best against the many objections given in the literature to extant philosophical analyses of bullshitting.

While these further conceptual questions must await thorough treatment in future work, I suspect that large language models have potential not only to *be*—and then *stop being*—bullshitters but also to shed light on the phenomenon of bullshitting itself. This is an increasingly urgent project. In a world where the sheer volume of verbal output cascading through our information environments each day makes disingenuous speech ever harder to spot and challenge, the ability to call bullshit—whether on humans or machines—is essential. I hope to have begun making that task somewhat more tractable.

## Data Availability

No data was gathered.

## Footnotes

1 See Cohen (2002) and Frankfurt (2002) for discussion of these questions.

2 For overviews, see Carson (2016) and Stokke (2018a).

3 See Fallis (2009, 2012), Dynel (2011), Briciu (2021).

4 See Stokke & Fallis (2016Stokke, 2018a, b; Fallis, (2015).

5 See Kenyon and Saul (2022).

6 See, for example, Carson (2016).

7 Hicks et al.'s (2024) article, entitled “ChatGPT is bullshit,” appeared after the current one was submitted for consideration. Since their argument is highly relevant to mine, I included discussion of it during the review process (see Sect. 3).

8 For helpful primers on large language models, see Wolfram (2023b); Lee and Trott (2023); Millière & Buckner (2024). I note that Shanahan et al. (2023) are careful to refer to applications like ChatGPT as “dialogue agents,” distinguishing them from the large language models on which they are built. For my purposes here, however, I collapse that distinction and use “large language models” across the board.

9 To keep things simple, I will tend to talk as if the linguistic units operated on are *words*. Thank you to a reviewer for encouraging me to be explicit about this.

10 For recent surveys of possible applications, see Kaddour et al. (2023); Hadi et al. (2024).

11 See, for example, Alkaissi and McFarlane (2023), Emsley (2023).

12 As when ChatGPT appeared to accuse Marietje Schaacke of being a terrorist, in a case reported widely in August 2023.

13 Thanks also to a reviewer for encouraging me to address this view.

14 See Grice (1989, especially Chaps. 5 and 6) for a canonical discussion. Admittedly, on some uses of “say” or “utter” what is said or uttered may depend more on linguistic conventions than speaker intentions—for further discussion, see Borg and Fisher (2021). Thus, we might sensibly talk about what is said by a large language model as something which corresponds to the conventional meaning of its outputted sentences. However, since Hicks et al. are primarily concerned with the activity of bullshitting, not the resulting content, they are concerned with the properties of *speakers* of utterances (specifically, whether or not those speakers have attitudes towards the truth or falsity of what they say). Therefore, the authors need to establish that large language models can be speakers who can say things, if the models are to be capable of bullshitting.

15 A similar argument could be run with competing analyses of bullshitting, which equally assume that bullshitters are speakers who say things.

16 For arguments that the outputs of large language models are meaningful in the relevant sense, see Borg (forthcoming) and Mandelkern and Linzen (2023). For a contrary view, see Mallory (2023).

17 John Major is a British political figure who served as prime minister from 1990 to 1997. I was not aware of any incident involving his falling in a pond.

18 In fact, this seems not to be the case, based on anecdotal evidence from other users who have been able to generate false accounts in response to the same or similar prompts.

19 Thank you to reviewers for encouraging me to discuss these initiatives.

20 One could even imagine a model that assesses its outputs for truth or falsity and only releases those deemed to be *false*, rather than those deemed to be *true* (albeit it is hard to think of a use case for such a system). Perhaps it would then be doing something akin to human *lying*. Whatever its other epistemic failings, however, it would not count as bullshitting.

## Code availability

N/A.
